# Relationship between type A personality and coronary heart disease

**DOI:** 10.1192/j.eurpsy.2022.950

**Published:** 2022-09-01

**Authors:** W. Saadi, M. Gorgi, N. Fouel, N. Haloui, W. Belaguide, W. Abdelghaffar, M.I. Bouzid, R. Rafrafi

**Affiliations:** 1 Mongi Slim hospital, Psychiatry, tunis, Tunisia; 2 Taher Sfar Hospital, Psychiartric Department, Mahdia, Tunisia; 3 Faculty of medicine of Tunis University Tunis El Manar, Psychiatry, Tunis, Tunisia

**Keywords:** Type A personality, coronary heart disease

## Abstract

**Introduction:**

Since the work of R.H. Rosenman and Meyer Friedman in 1959, a correlation has been established between type A behavioral patterns and the occurrence of coronary heart disease. Type A personality has been found to be more of a coronary risk factor than a poor prognostic factor once coronary disease has set in. Subsequent studies have not supported such a relationship.

**Objectives:**

The objective of our work was to investigate the association between type A personalities and coronary heart disease.

**Methods:**

We conducted a retrospective study involving a sample of 200 patients recruited at the Mohamed Tahar Maâmouri Hospital in Nabeul. Our sample was composed of 100 coronary patients hospitalized or followed as outpatients in the cardiology department and 100 controls hospitalized or followed as outpatients in the general surgery or orthopedics department respectively. The study was conducted between April 15 and June 30, 2014. Personality type A was assessed according to the Bortner questionnaire.

**Results:**

After performing a binary logistic regression to adjust for the associations looked for, and taking into account confounding factors, we did not observe a statistically significant association between type A personality and coronary pathology (p=0.123). In addition, type A personality was significantly associated with the following factors: diabetes (p=0.040), hypertension (p=0.049), and age <49 years (p=0.002) in coronary heart disease.

**Conclusions:**

Future large-scale, multicenter, longitudinal studies with follow-up over time of patients would be necessary to consolidate our findings.

**Disclosure:**

No significant relationships.

